# Gastroesophageal Reflux Disease and Hiatal Hernia After Laparoscopic Sleeve Gastrectomy: A Retrospective Cohort Study

**DOI:** 10.7759/cureus.23024

**Published:** 2022-03-10

**Authors:** Bandar F Almutairi, Abdullah B Aldulami, Nizar M Yamani

**Affiliations:** 1 College of Medicine, Prince Sattam Bin Abdulaziz University, Al-Kharj, SAU; 2 College of Medicine, King Faisal University, Al-Ahsa, SAU; 3 Surgery, King Abdulaziz Medical City, Riyadh, SAU

**Keywords:** laparoscopic sleeve gastrectomy, saudi arabia, incidence, hiatal hernia, gerd

## Abstract

Introduction: Laparoscopic sleeve gastrectomy (LSG) has shown good results in terms of weight loss and improvement of obesity-comorbidities, even though its effect on inducing new-onset gastroesophageal reflux disease (GERD) is still a matter of debate. This study aims to estimate the incidence of GERD and hiatal hernia post LSG and to identify associated risk factors of GERD development.

Methods: This is a retrospective cohort study of all patients who underwent LSG surgery at the National Guard medical hospitals (Riyadh and Al-Ahsa) between January 2016 and February 2019. Patients who had undergone LSG, who had a history of GERD or hiatal hernia preoperatively, or who had intraoperative hiatal hernia repair were excluded. Mean, standard deviation, and independent t-test was used for numerical variables, while frequencies, percentages, and chi-square test were used for categorical variables.

Results: There were 142 patients included in this study, with the mean age being 39,38 ± 12.68 years, and 64.8% of patients were female. Patients were followed up for 24 months. The incidence of GERD post-operation was 33.% (n=47) and hiatal hernia was 3.5% (n=5). Significantly associated risk factor for post-operative GERD were as follows: age (p=0.026), gender (p=0.038), and hypertension (p=0.014).

Conclusion: Incidence of GERD was shown to be relatively high, while hiatal hernia was low; besides age, gender and hypertension, none of the other variables was associated with the development of GERD.

## Introduction

Obesity has become a pandemic issue all over the world with a high prevalence reaching 650 million of the world’s adult population [1]. According to a survey conducted in the Saudi population, it showed that 27.8% were obese with females having a high prevalence more than males of 33.5 vs. 24.1%, respectively [2]. Obesity has been associated with the development of many comorbidities including hypertension, dyslipidemia, type 2 diabetes mellitus, stroke, and coronary heart disease (CHD) [3]. Obesity also is known to be an independent risk factor for the development of gastroesophageal reflux disease (GERD) and hiatal hernia [4]. Besides diet, lifestyle modifications, and pharmacotherapy, surgical management of obesity is highly effective and comes with good results in losing weight. Bariatric surgeries have been increasing in the last decade. In 2013, 0.01% of the world's population underwent bariatric surgery [5]. A variety of surgical options are available to deal with obesity; laparoscopic sleeve gastrectomy (LSG) has become more popular among other procedures. It accounts for 47.0% of all bariatric surgeries [6]. In Saudi Arabia, the most preferred bariatric surgery is LSG; in 2013, 13,194 surgeries were performed, 10,502 of them were LSG [6]. It is considered one of the most valuable options for treating obesity. When compared with nonsurgical intervention, it showed effectiveness in losing weight and great improvement of obesity comorbidities [7]. A systematic review that studied the relationship between bariatric surgery and GERD showed that it helped in the improvement of GERD symptoms with laparoscopic roux-en-Y gastric bypass (LYRGB) having a more favorable effect in the improvement of GERD symptoms than LSG [8]. LSG has shown heterogeneity in terms of GERD outcomes.

A retrospective single-center study conducted by Chopra et al. showed an improvement of GERD symptoms post LSG of 45.92% [9], the same result has also been found in the study by Rawlins et al. where it results in 53% resolution and improvement of symptoms [10]. In contrast, prospective studies conducted by Burgerhart et al. and Pallati et al. have shown worsening of GERD symptoms post-LSG of 43% and 4.6%, respectively [11,12]. In addition, LSG itself might also increase the incidence of new-onset GERD post-LSG. One study demonstrated a 27.5% incidence of GERD after LSG [13]; another study that was conducted on 109 patients who underwent LSG showed that 27 out of 73 patients had developed de novo GERD symptoms after LSG which accounted for 36.9% [14]. Other studies also reported the same findings [15-17]. It is sensible to conclude that the results on the effect of LSG on GERD are conflicting and it is effect on inducing a new-onset GERD is still a matter of debate. There are multiple proposed mechanisms for increased GERD prevalence post-LSG. Angle of His is known to play a protective role against GERD development [18], and its disruption during LSG could contribute to a rise in GERD symptoms [19]. The decrease of lower esophageal sphincter pressure following LSG has also been proposed as a cause of the appearance of GERD symptoms [20]. Himpens et al. have described a formation of an anatomic change in LSG patients called “neofundus” leading to mid-stomach stenosis and it causes stasis of food and increased acid production and that in turn leads to reflux symptoms [21]. However, the relationship between developing GERD and hiatal hernia after LSG remains unclear. In this study, we aim to investigate the incidence of new-onset GERD and hiatal hernia after LSG and to identify any associated risk factors for the development of GERD.

## Materials and methods

Study design and subjects

This retrospective cohort study was conducted at the National Guard medical hospitals: King Abdulaziz Medical City (KAMC) in Riyadh and King Abdulaziz Hospital (KAH) in Al-Ahsa from January 2016 to February 2019. We designed this study to investigate the incidence rate of GERD and hiatal hernia after LSG and to identify any possible risk factors for the development of GERD postoperatively. This study was performed with the approval of the Institutional Review Board of King Abdullah International Medical Research Center (KAIMRC) at the Ministry of National Guard Health Affairs (NGHA) in accordance with international research ethics standards (IRBC/1167/19). Inclusion criteria consisted of all patients who underwent LSG in KAMC (Riyadh) or KAH (Al-Ahsa) from January 2016 to February 2019. The exclusion criteria consisted of any patient who had the following: previous bariatric surgery, a history of GERD symptoms or diagnosed with GERD before surgery, diagnosed with hiatal hernia preoperatively, or had intraoperative hiatal hernia repair.

Data collection

We followed patients for a period of 24 months; we used BestCare electronic medical records (EHRs) system to access patients’ medical records to extract the patient’s data in terms of age, gender, obesity-related comorbidities such as hypertension, diabetes mellitus, dyslipidemia, and obstructive sleep apnea, smoking status, height, pre- and post-operative BMI and excess BMI loss percentage (EBMIL%). For a diagnosis of GERD or hiatal hernia postoperatively, GERD symptoms grade are classified clinically as follows: none; grade 1: mild symptoms and no proton pump inhibitor (PPI) use; grade 2: moderate symptoms and periodic PPI use; grade 3: severe symptoms and frequent PPI use, upper endoscopy, esophageal manometry and 24-hours PH mentoring, barium contrast study, and the period from surgery at which GERD had been diagnosed.

Statistical analysis

Data were collected in Microsoft Excel (Microsoft Corp, Redmond, WA) and converted to SPSS, version 25.0 (SPSS Inc, Chicago, IL). Numerical variables were described as mean and standard deviation, while the categorical variables were described as frequencies and percentages. Independent t-test was used for comparisons between the GERD and non-GERD groups with respect to all numerical variables, while chi-square test was used with respect to all categorical variables. A p-value of <0.05 was considered significant.

## Results

From January 2016 to February 2019, a total of 500 patients underwent laparoscopic sleeve gastrectomy at NGHA institutions, 142 met the inclusion criteria and were included in the study. The mean age was 39.38 ± 12.68 years, males accounted for 35.2% (n=50) while females were 64.8% (n=92). The percentage and frequency of comorbidities were as follows: hypertension 33.09% (n=47), diabetes mellitus 32.4% (n=46), dyslipidemia 24.6% (n=35), obstructive sleep apnea 16.2% (n=23); smokers accounted for 14.1% (n=20) Baseline characteristics of the patients are presented in Table 1. Preoperative BMI mean was 45.29 ± 7.22, post-operative BMI mean at six months was 34.40 ± 6.30 with a mean change in BMI of 11.91 kg/m2, height, and EBMI% also shown in Table 1. Of the 142 patients who were followed up for 24 months, post-operative new-onset GERD was observed in 47 (33.1%) patients, of those 29 (61.7%) reports mild symptoms with no PPI use, 13 (27.65%) reported moderate symptoms with periodic PPI use, and five (10.63%) reported severe symptoms with frequent PPI use. Fifteen patients were diagnosed using upper endoscopy, while 32 were diagnosed by symptoms reporting and PPI use. Post-operative hiatal hernia was observed in five (3.5%) patients only (Table 2). It was significantly associated with GERD (p=0.007). The average time for the development of GERD was six months, and most cases presented three months after the procedure. Out of the 47 patients who developed GERD, 36 were females, and 11 were males; gender was significantly associated with GERD development (p=0.038), the mean age was 42.74 ± 11.72 years and it was also significantly associated with GERD development (p=0.026). Out of the comorbidities (hypertension, diabetes mellitus, dyslipidemia, obstructive sleep apnea) and smoking, hypertension was the only statistically significant associated risk factor (n=22, p=0.014) (Table 3). Preoperative and postoperative BMI were not statistically significant (p=0.440, p=0.549), nor the EBMI% (p=0.419) (Table 3).

**Table 1:** Baseline characteristics of the patients. SD: standard deviation; N: number

Variable	N (%) / MEAN ± SD	
Age (years), mean ± SD	39.38 ± 12.68	
Gender, n (%)	Male	50 (35.2%)
	Female	92 (64.8%)
Hypertension, n (%)	Yes	47 (33.09%)
	No	95 (66.9%)
Diabetes mellitus, n (%)	Yes	46 (32.4%)
	No	96(67.6%)
Dyslipidemia, n (%)	Yes	35 (24.6%)
	No	107 (75.4%)
Obstructive sleep apnea, n (%)	Yes	23 (16.2%)
	No	119 (83.8%)
Smoking, n (%)	Yes	20 (14.1%)
	No	122 (85.9%)
Height (cm), mean ± SD	161.60 ± 8.94	
Pre-operative measurement (kg/m^2^), mean ± SD	Pre-BMI	45.29 ± 7.22
Post-operative measurement (kg/m^2^), mean ± SD	Post-BMI at 6 Months	34.40 ± 6.30
	excess body mass index loss (EBMIL %)	0.25 ± 0.06

**Table 2:** Incidence of GERD and hiatal hernia. GERD: gastroesophageal reflux disease; PPI: proton pump inhibitor; N: number

Variable	N (%)	
GERD	Yes	47 (33.1)
	No	95 (66.9)
GERD symptoms category	Grade 1 (mild symptoms, no PPI use)	29 (61.7)
	Grade 2 (moderate symptoms, periodic PPI use)	13 (27.65)
	Grade 3 (severe symptoms, frequent PPI use)	5 (10.63)
Hiatal hernia	Yes	5 (3.5)
	No	137 (96.5)

**Table 3:** Association between GERD and possible risk factors. SD: standard deviation; N: number

Variable	GERD N (%) \ MEAN ± SD		P value
	Yes	No	
Age	42.74 ± 11.72	37.72 ± 12.86	0.026
Gender			0.038
Male	11 (22.0%)	39 (78.0%)	
Female	36 (39.1%)	56 (60.9%)	
Hypertension			0.014
Yes	22 (46.8%)	25 (55.3%)	
No	25 (26.31%)	70 (73.68%)	
Diabetes mellitus			0.150
Yes	19 (41.3%)	27 (58.7%)	
No	28 (29.2%)	68 (70.8%)	
Dyslipidemia			0.863
Yes	12 (34.3%)	23 (65.7%)	
No	35 (32.7%)	72 (67.3%)	
Obstructive sleep apnea			0.441
Yes	8 (20.0%)	15 (14.71%)	
No	32 (80.0%)	87 (85.29%)	
Smoking			0.406
Yes	5 (25.0%)	15 (75.0%)	
No	42 (34.4)	80 (65.6%)	
Pre-operative measurement			0.440
Pre-BMI (kg/m^2^)	45.96 ±7.92	44.96 ± 6.87	
Post-operative measurement			
Post-BMI (kg/m^2^)	34.81 ± 6.57	34.07 ± 6.11	0.549
excess body mass index loss (EBMIL %)	0.24 ± 0.06	0.25 ± 0.06	0.419

## Discussion

Bariatric surgeries have gained popularity worldwide, with LSG being the preferred method of losing weight, as LSG alone accounts for 47.0% of all bariatric surgeries [6]. Although LSG showed effectiveness in weight loss and resolution of obesity-related comorbidities, there has been considerable debate and controversy over the effect of LSG on increasing the incidence of de novo GERD postoperatively. In this study, we aim to investigate the incidence of new-onset GERD and hiatal hernia after LSG.

We report an incidence of new-onset GERD post-LSG of 33.1% during a follow-up period of 24 months. This incidence rate is similar to previous studies that determined the incidence of new-onset GERD to be 34.9% and 36% respectively [15,22]; the overall reported incidence in previous studies ranged from 2.1% to 47.06% [16,17]. In a meta-analysis comprising 46 studies, the authors conclude the incidence of de novo reflux was 23% [23]. We believe this discrepancy in the incidence rates is due to the lack of standard evaluation methods to diagnose GERD. In our study, they used a combination of objective and subjective methods, as half of the patients were diagnosed using upper endoscopy while the others by symptoms and PPI trial. In contrast, other studies rely only on questionnaires or symptoms reporting as their way of diagnosing GERD [9,16,17], though symptoms by themselves are an unreliable way of diagnosing GERD [24], and it might overestimate its real incidence. Due to this reason, the need for more reliable tests such as upper endoscopy, or esophageal function tests such as esophageal manometry and 24-hours PH mentoring (even though the latter has no utility in routine clinical settings) offer a more precise estimate of the incidence.

These high incidences might exclude LSG as a preferred method for losing weight and demand the need for effective procedures. A systematic review has concluded that LRYGB had a lower incidence compared to LSG in the development of new-onset GERD [8]. Another study also found that LRYGB carries a low incidence of GERD development compared to LSG with an incidence rate of 16% to 67%, respectively [25], although LSG might have a better chance as concluded by a study that demonstrated that it carries less incidence when it is performed under careful attention to the surgical technique [26]. 

Multiple mechanisms have been described that might influence the development of de novo GERD after LSG, including disruption of the angle of His [19], neofundus [21], and decreased lower esophageal sphincter pressure [20]. Of note, the formation of a de novo hiatal hernia has also been described as a proposed mechanism [27,28]. In our cohort, the incidence of de novo hiatal hernia was 3.5% and it was significantly associated with GERD (p=0.007); one study reported a similar incidence of 5.7% [29], although another study reported a rate up to 72.5%; they attributed their high incidence to the surgical exploration method they used for diagnosing hiatal hernia [30], In our findings and the finding reported by Braghetto et al., [29], the diagnosis was based on upper endoscopy. It is difficult to identify the mechanism involved in the development of de novo hiatal hernia; multifactorial mechanisms might be responsible mainly during the creation of the gastric tube with the dissection of the angle of His and the left pillar and the dissection of phrenogastric and pharyngoesophageal ligaments. Other mechanisms that might also have a role are rapid weight loss and weight regain, negative chest pressure, the tube-shaped gastric sleeve and the difficulty of fixing it to the surrounding structures [30].

We set out to determine the associated risk factors for the development of GERD after LSG. As shown in one study, higher age was found to be associated with a higher incidence of GERD [25]. Likewise, we did find a significant association between age and GERD with a higher mean age in patients who developed GERD. Other authors did not find any association between age and GERD development after LSG [17,29]. Gender was a statistically associated risk factor for GERD; in our study, interestingly, females showed a higher association than males, although that might be explained in part due to the large number of females in our study. Gender was not found to be significantly associated with GERD in the study by Althuwaini et al. [17]. Hypertension was also identified to be significantly associated with GERD development, though it can be interpreted as the result of chance, as there is no physiological explanation for such an association. However, further research is needed to explore this finding. Pre-operative BMI was found to be significantly associated in the study by Althuwaini et al. [17]. As for our study, we did not find an association between preoperative BMI and GERD. Postoperative BMI was also not a significant risk factor for GERD. 

There were some limitations in the present study. First, this is a retrospective study with data retrieved by medical record review, therefore, we did not have full control of the diagnosing methods. The second limitation was that our study was supposed to be conducted at three centers, unfortunately, we were not able to access the database of the third center, and this limited the number of patients on our study. However, most of the studies we reviewed had a similar number of patients to ours. 

## Conclusions

In this study, up to one-third of the patients followed during 24 months after LSG developed GERD; most of the patients reported their symptoms within the first six months. This research also observed that the incidence of hiatal hernia after bariatric surgery is low. Female gender was a significant risk factor. In addition, patients affected by GERD were seen to have a significantly higher mean age. Hypertension was also a risk factor for the development of GERD. In contrast, our study revealed that other comorbidities (diabetes mellitus, dyslipidemia, obstructive sleep apnea), smoking, pre- and post-operative BMI, and excess BMI loss, were not significant risk factors for GERD. We recommend offering consultation for patients with identified risk factors about the risk of having GERD and the need for alternative methods to lose weight, such as LRYGB. Future randomized and multi-center trials are needed so that we can understand the anatomic and pathophysiological mechanisms to put an end to this debate and to delineate the risk factor associated with GERD development after LSG.

## References

[1] (2021). Obesity and overweight. https://www.who.int/news-room/fact-sheets/detail/obesity-and-overweight..

[2] Memish ZA, El Bcheraoui C, Tuffaha M (2014). Obesity and associated factors--Kingdom of Saudi Arabia, 2013. Prev Chronic Dis.

[3] Pi-Sunyer X (2009). The medical risks of obesity. Postgrad Med.

[4] Soricelli E, Casella G, Rizzello M, Calì B, Alessandri G, Basso N (2010). Initial experience with laparoscopic crural closure in the management of hiatal hernia in obese patients undergoing sleeve gastrectomy. Obes Surg.

[5] Angrisani L, Santonicola A, Iovino P, Formisano G, Buchwald H, Scopinaro N (2015). Bariatric surgery worldwide 2013. Obes Surg.

[6] Himpens J, Ramos A, Welbourn R, Dixon J, Kinsman R, Walton P (2018). Fourth IFSO Global Registry Report. https://www.ifso.com/pdf/4th-ifso-global-registry-report-last-2018.pdf.

[7] Gloy VL, Briel M, Bhatt DL (2013). Bariatric surgery versus non-surgical treatment for obesity: a systematic review and meta-analysis of randomised controlled trials. BMJ.

[8] Gu L, Chen B, Du N (2019). Relationship between bariatric surgery and gastroesophageal reflux disease: a systematic review and meta-analysis. Obes Surg.

[9] Chopra A, Chao E, Etkin Y, Merklinger L, Lieb J, Delany H (2012). Laparoscopic sleeve gastrectomy for obesity: can it be considered a definitive procedure?. Surg Endosc.

[10] Rawlins L, Rawlins MP, Brown CC, Schumacher DL (2013). Sleeve gastrectomy: 5-year outcomes of a single institution. Surg Obes Relat Dis.

[11] Burgerhart JS, Schotborgh CA, Schoon EJ, Smulders JF, van de Meeberg PC, Siersema PD, Smout AJ (2014). Effect of sleeve gastrectomy on gastroesophageal reflux. Obes Surg.

[12] Pallati PK, Shaligram A, Shostrom VK, Oleynikov D, McBride CL, Goede MR (2014). Improvement in gastroesophageal reflux disease symptoms after various bariatric procedures: review of the Bariatric Outcomes Longitudinal Database. Surg Obes Relat Dis.

[13] Braghetto I, Csendes A, Lanzarini E, Papapietro K, Cárcamo C, Molina JC (2012). Is laparoscopic sleeve gastrectomy an acceptable primary bariatric procedure in obese patients? Early and 5-year postoperative results. Surg Laparosc Endosc Percutan Tech.

[14] Viscido G, Gorodner V, Signorini F, Navarro L, Obeide L, Moser F (2018). Laparoscopic sleeve gastrectomy: endoscopic findings and gastroesophageal reflux symptoms at 18-month follow-up. J Laparoendosc Adv Surg Tech A.

[15] Gorodner V, Buxhoeveden R, Clemente G, Solé L, Caro L, Grigaites A (2015). Does laparoscopic sleeve gastrectomy have any influence on gastroesophageal reflux disease? Preliminary results. Surg Endosc.

[16] Arias E, Martínez PR, Ka Ming Li V, Szomstein S, Rosenthal RJ (2009). Mid-term follow-up after sleeve gastrectomy as a final approach for morbid obesity. Obes Surg.

[17] Althuwaini S, Bamehriz F, Aldohayan A (2018). Prevalence and predictors of gastroesophageal reflux disease after laparoscopic sleeve gastrectomy. Obes Surg.

[18] Lortat-Jacob JL, Robert F (1953). Malpositions of the cardia and greater curvature [Article in French]. Arch Mal Appar Dig Mal Nutr.

[19] Himpens J, Dapri G, Cadière GB (2006). A prospective randomized study between laparoscopic gastric banding and laparoscopic isolated sleeve gastrectomy: results after 1 and 3 years. Obes Surg.

[20] Braghetto I, Lanzarini E, Korn O, Valladares H, Molina JC, Henriquez A (2010). Manometric changes of the lower esophageal sphincter after sleeve gastrectomy in obese patients. Obes Surg.

[21] Himpens J, Dobbeleir J, Peeters G (2010). Long-term results of laparoscopic sleeve gastrectomy for obesity. Ann Surg.

[22] Tai CM, Huang CK, Lee YC, Chang CY, Lee CT, Lin JT (2013). Increase in gastroesophageal reflux disease symptoms and erosive esophagitis 1 year after laparoscopic sleeve gastrectomy among obese adults. Surg Endosc.

[23] Yeung KTD, Penney N, Ashrafian L, Darzi A, Ashrafian H (2022). Does sleeve gastrectomy expose the distal esophagus to severe reflux?: A systematic review and meta-analysis. Ann Surg.

[24] Galvani C, Fisichella PM, Gorodner MV, Perretta S, Patti MG (2003). Symptoms are a poor indicator of reflux status after fundoplication for gastroesophageal reflux disease: role of esophageal functions tests. Arch Surg.

[25] Navarini D, Madalosso CA, Tognon AP, Fornari F, Barão FR, Gurski RR (2020). Predictive factors of gastroesophageal reflux disease in bariatric surgery: a controlled trial comparing sleeve gastrectomy with gastric bypass. Obes Surg.

[26] Daes J, Jimenez ME, Said N, Daza JC, Dennis R (2012). Laparoscopic sleeve gastrectomy: symptoms of gastroesophageal reflux can be reduced by changes in surgical technique. Obes Surg.

[27] Baumann T, Grueneberger J, Pache G (2011). Three-dimensional stomach analysis with computed tomography after laparoscopic sleeve gastrectomy: sleeve dilation and thoracic migration. Surg Endosc.

[28] Oor JE, Roks DJ, Ünlü Ç, Hazebroek EJ (2016). Laparoscopic sleeve gastrectomy and gastroesophageal reflux disease: a systematic review and meta-analysis. Am J Surg.

[29] Braghetto I, Korn O (2019). Late esophagogastric anatomic and functional changes after sleeve gastrectomy and its clinical consequences with regards to gastroesophageal reflux disease. Dis Esophagus.

[30] Saba J, Bravo M, Rivas E, Fernández R, Pérez-Castilla A, Zajjur J (2020). Incidence of de novo hiatal hernia after laparoscopic sleeve gastrectomy. Obes Surg.

